# The trends of mortality and years of life lost of cancers in urban and rural areas in China, 1990‐2017

**DOI:** 10.1002/cam4.2765

**Published:** 2019-12-24

**Authors:** Xingzu Cen, Dongming Wang, Weiwei Sun, Limin Cao, Zhuang Zhang, Bin Wang, Weihong Chen

**Affiliations:** ^1^ Department of Occupational & Environmental Health School of Public Health Tongji Medical College Huazhong University of Science and Technology Wuhan China; ^2^ Key Laboratory of Environment and Health Ministry of Education & Ministry of Environmental Protection, and State Key Laboratory of Environmental Health (Incubating) School of Public Health Tongji Medical College Huazhong University of Science and Technology Wuhan China; ^3^ Examining Division for Materials Engineering Inventions China National Intellectual Property Administration Beijing China

**Keywords:** burden of disease, cancer, mortality rate, trend, years of life lost

## Abstract

**Background:**

With the rapid development of the socioeconomic status, the mortality of several cancers has been changed in China during the past 30 years. We aimed to estimate the trends of mortality and years of life lost (YLLs) of various cancers in urban and rural areas of China from 1990 to 2017.

**Methods:**

The mortality data were collected from Chinese yearbooks and the age structure of population from the Chinese sixth population census were used as reference to calculate age‐standardized mortality rates (ASMRs) and YLLs rates. Joinpoint regression analysis was implemented to calculate the annual percent change (APC) of mortality rates and YLL rates for cancers. YLLs owing to premature death were calculated as age‐specific cancer deaths multiplied by the reference life expectancy at birth of 80 years for male and 82.5 years for female.

**Results:**

The ASMRs of all cancers showed significant decreasing trends for urban residents from 1990 to 2017, such downward trend without significance was also observed among rural residents. Interestingly, ASMRs of lung cancer and breast cancer have raised continuously in rural areas since 1990. The age‐standardized YYL rates for urban and rural residents decreased with 1.02% and 0.85% per year, respectively. YLLs in rural areas were higher than those in urban areas, whereas YLLs of urban outstripped those of rural finally with the increasing in YLLs of urban areas (216.71% for men and 207.87% for women).

**Conclusion:**

The ASMRs and YLL rates of all cancers have declined in urban and rural areas from 1990 to 2017. YLLs increased in urban areas and remained higher level in rural areas after 2014 year. Preventive measures should be strengthened to against cancer, especially for lung cancer.

## Graphical abstract

Mortality rate and YLL rate of cancer were analyzed by joinpoint regression. The ASMRs of cancer in both urban and rural areas in China declined from 1990 to 2017. YLLs increased in urban areas and remained higher levels in rural areas from 1990 to 2017.

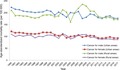

## 1 INTRODUCTION

It is reported that cancer is the second leading cause of deaths worldwide, only behind cardiovascular diseases.^1^, ^2^ There were 17.5 million cancer cases globally and 8.7 million deaths reported by Global Burden of Disease Study (GBD) 2015.^2^, ^3^ According to the GBD 2017, deaths from cancers increased by 25.4% between 2007 and 2017, from 7.62 million deaths in 2007 to 9.56 million deaths in 2017. Age‐standardized mortality rates (ASMRs) due to cancers decreased by 4.4% from 2007 to 2017, whereas years of life lost (YLLs) increased by 19.6%.^1^ In China, cancers caused 24.94% (2.61 million) of all deaths in 2017, which increased by 83.89% when compared with those in 1990. And cancers caused 63.25 million disability‐adjusted life years (DALYs) in China, of which 96.88% come from YLLs and 3.12% come from years lived with disability (YLDs).^4^ Furthermore, lung, liver, and gastric cancer contributed much more to YLLs in 2017 in comparison with those in 1990, which ranked the third, fifth, seventh of causes of YLLs for both sexes in 2017, respectively.^5^ As one of the most common noncommunicable diseases (NCDs), cancer has induced enormous burdens to society with the population aging, transition in lifestyles toward high‐risk behaviors and environment worsening.^6^

China has experienced a rapid industrialization and socioeconomic transition in the past three decades, resulting in several changes in cancer risk factors.^7^ Urbanization, aging, and improvements of medical service condition have led to a shift in the disease burden from infectious diseases to noncommunicable diseases.^6^, ^8^ Nowadays, environmental pollutions resulting from rapid industrialization are becoming more serious. Air quality in several cities of China is also terrible, and exposure to air pollutants is believed to increase the risk for lung cancer.^9^, ^10^ Moreover, unhealthy lifestyle and diet pattern related to cancer have also changed, the burden of diseases attributable to individual behaviors and practices is rising.^6^, ^11^, ^12^ Risk factors, including smoking, infections, alcohol drinking, low fruit/vegetable intake, excess body weight, and physical inactivity, caused nearly half of cancer deaths in China in 2013.^13^ At the same time, medical service systems and dietary nutrition have been greatly improved, deaths from infectious diseases showed significant reduction and the life expectancy at birth increased by 8.4 years from 1990 to 2017 in China.^8^, ^14^ These factors mentioned above may be involved in the development of cancer during the last three decades. Therefore, it is essential to estimate the trends on the mortality and disease burden of cancers for cancer prevention and control.

Thus, our study aimed to analyze the level and trend of cancer mortality separated by cause, age, and sex in China from 1990 to 2017. And we focused on comparing the difference of mortality rates and YLLs between urban and rural areas, which could provide evidences for decision makers to prioritize resources and implement preventive measures to against cancer.

## 2 MATERIALS AND METHODS

### 2.1 Data sources

Demographic data were obtained from officially published reports, including China Population Statistics Yearbook (1991‐2006) and China Population and Employment Statistics Yearbook (2007‐2018), which contains the demographic composition of gender and age in urban and rural regions. These data were obtained through sample surveys and census, and we calculated the status of the entire population combining with the sampling ratio. The data on cause and age‐specific mortality were collected from Chinese Health Statistical Annual Report (1991‐2002), China Health Statistics Yearbook (2003‐2012), China Health and Family Planning Statistics Yearbook (2013‐2017), and China Hygiene and Health Statistics Yearbook (2018), which includes mortality rates caused by different factors for 20 age groups and both sexes in urban and rural areas. Urban and rural areas is distinguished according to administrative districts, urban areas include municipalities and prefecture‐level cities, and rural areas include counties and county‐level cities. Cancers were classified according to the International Classification of Diseases (ICD) codes, Ninth Revision (ICD‐9, 1990‐2001), and Tenth Revision (ICD‐10, 2002‐2017).

### 2.2 Statistical analysis

The mortality rates (per 100 000 people) were calculated through direct age standardization with Chinese sixth population census in 2010 as the standard population for each age group from 1990 to 2017.^15^ Joinpoint regression was used to analyze the trend of mortality rates during the study period through calculation of annual percent change (APC). The maximum number of joinpoints was set to three. Joinpoint regression analysis was performed by Joinpoint Regression Program 4.5.0.1. In addition, 95% confidence interval (CI) for APC was calculated and the *P* value of less than .05 was considered statistically significant.

YLLs due to premature death were computed by the number of deaths at each age multiplied by a standard life expectancy at that age.^16^ We chose the standard life expectancy at birth fixed at 82.5 years for female and 82.0 years for male.^17^ The average age at death was set to the mid‐point of each age group, except for people over 85 years old in whom it was assumed to be 87.5 years.^17^, ^18^ Total YLLs were the sum of YLL at each age group. The method of calculation in age‐standardized YLL rates was similar to ASMRs. And the trend on YLL rate was also analyzed by joinpoint regression.

## 3 RESULTS

### 3.1 Trends on cancer mortality rates

**Figure 1:**
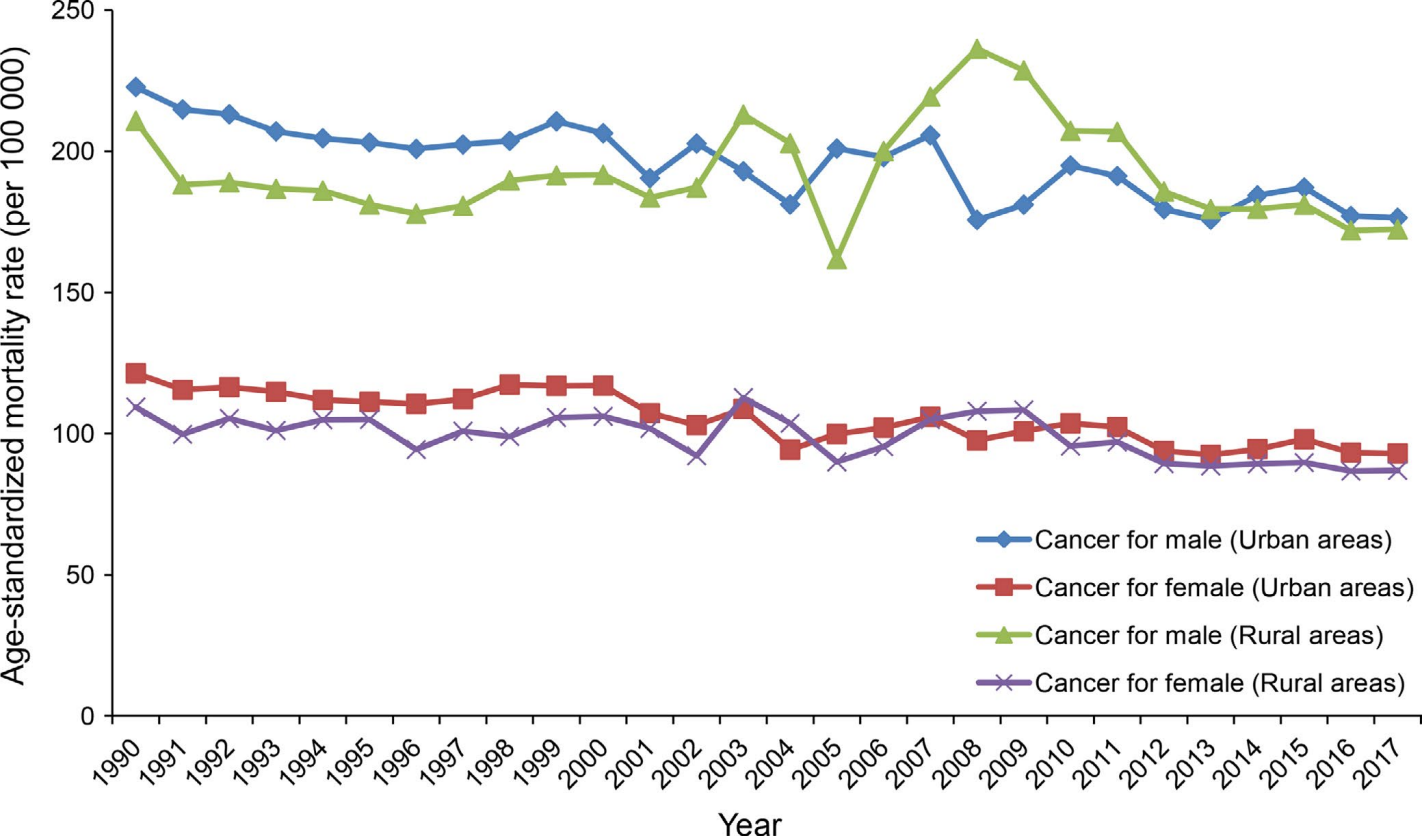
The trend of the age‐standardized mortality rates of cancer for male and female in urban areas and rural areas from 1990 to 2017

The trends on ASMRs of cancers among urban and rural residents from 1990 to 2017 in China are shown in Figure 1. Compared with female residents, the ASMRs of males were significantly higher in both urban and rural areas. The ASMRs of men in urban areas were slightly higher than those in rural areas except for 2003 and 2004, and from 2006 to 2013. The difference of ASMRs between urban women and rural women has narrowed gradually from 1990 to 2017. The ASMRs of urban residents continued to decline slowly, which ranged from 222.79 to 176.45 deaths per 100 000 people with 20.80% decrease for men, and from 121.37 to 92.94 deaths per 100 000 people with 23.42% falling for women between 1990 and 2017, respectively. The ASMRs ranged from 210.88 to 172.37 deaths per 100 000 people for men and from 109.41 to 86.95 per 100 000 people for women in rural areas during the study period.

Table 1 shows ASMRs of various cancers among urban and rural residents ranged from 1990 to 2017, as well as average APC and 95% CI of the study period (28 years) and APC for each sub‐period. In urban areas, the ASMRs of cancer were 168.09 and 133.27 deaths per 100 000 people in 1990 and 2017, respectively, with significant reduction by 0.73% (95% CI: −0.89% to −0.57%) per year during the whole period. The ASMRs of leukemia, lung, liver, gastric, esophagus, breast, nasopharyngeal, and bladder cancer showed significantly decreased trends from 1990 to 2017. However, we observed that individual cancer showed an increasing trend over a certain period of year. For example, the ASMR of cervical cancer increased with 9.61% per year from 2008 to 2017.

**Table 1:** Joinpoint analysis of the age‐standardized mortality rates from cancer in urban and rural areas from 1990 to 2017

	Mortality rate^a^ (1/100 000)		1990‐2017		Period 1		Period 2		Period 3		Period 4	
	1990	2017	AAPC (%)	95% CI	Years	APC (%)	Years	APC (%)	Years	APC (%)	Years	APC (%)
Cancer in urban areas	168.09	133.27	−0.73*	(−0.89, −0.57)	—							
Lung cancer	43.27	39.74	−0.36*	(−0.63, −0.08)	1990‐2000	0.64	2000‐2004	−5.8*	2004‐2007	4.81	2007‐2017	−0.12
Liver cancer	25.65	19.16	−1.07*	(−1.53, −0.61)	—							
Gastric cancer	28.78	14.31	−2.33*	(−2.76, −1.89)	—							
Esophagus cancer	13.31	8.99	−1.27*	(−1.88, −0.67)	1990‐2001	−2.56*	2001‐2005	8.03	2005‐2008	15.68	2008‐2017	2.40*
Colorectal cancer	11.05	10.81	−0.17	(−0.53, 0.18)	1990‐2000	1.01	2000‐2003	−7.90	2003‐2007	6.44	2007‐2017	−1.61*
Breast cancer b	4.26	4.12	−0.32*	(−0.61, −0.02)	—							
Leukemia	4.00	3.12	−0.53*	(−0.80, −0.25)	—							
Nasopharyngeal cancer	2.67	1.19	−3.01*	(−3.58, −2.45)	—							
Bladder cancer	2.65	1.74	−1.87*	(−2.31, −1.42)	1990‐2000	−0.55	2000‐2003	−12.71	2003‐2006	6.54	2006‐2017	−1.58*
Cervical cancer	2.03	2.18	−0.21	(−1.06, 1.50)	1990‐2008	−3.01*	2008‐2017	9.61*				
Cancer in rural areas	157.80	128.50	−0.17	(−0.51, 0.19)	—							
Lung cancer	21.29	35.22	2.61*	(2.18, 3.03)	—							
Liver cancer	34.19	22.37	−1.35*	(−1.87, −0.83)	1990‐2008	−0.09	2008‐2017	−4.74*				
Gastric cancer	36.91	17.01	−2.21*	(−2.69, −1.73)	1990‐2008	−1.12*	2008‐2017	−5.15*				
Esophagus cancer	29.64	10.49	−2.91*	(−3.93, −1.88)	1990‐2008	−0.74	2008‐2017	−8.63*				
Colorectal cancer	7.67	7.92	0.39	(−0.09, 0.87)	—							
Breast cancer c	2.06	3.08	1.16*	(0.40, 1.92)	—							
Leukemia	3.80	3.31	−0.22	(−0.49, 0.07)	1990‐2005	−0.93*	2005‐2008	6.12	2008‐2017	−2.15*		
Nasopharyngeal cancer	2.03	1.43	−2.14*	(−2.92, −1.34)	—							
Bladder cancer	1.39	1.37	−0.05	(−0.58, 0.49)	—							
Cervical cancer	2.50	2.53	−2.21*	(−3.70, −0.70)	1990‐2001	−0.39	2001‐2006	−16.71*	2006‐2017	7.79*		

Abbreviations: AAPC, average annual percent change; APC, annual percent change; CI, confidence interval.

*Note:*—, No joinpoints.

a Age‐standardized to the Chinese sixth population census in 2010.

b Female breast cancer in urban areas.

c Female breast cancer in rural areas.

* Significantly difference from zero (*P *< .05)

Among rural residents, the ASMR of cancer in 1990 was 157.80 per 100 000 people, and it dropped to 128.50 per 100 000 people in 2017 without significant difference. Significant downward trends of ASMRs were observed for liver, gastric, esophagus, nasopharyngeal, and cervical cancer in rural areas, whereas ASMRs of lung and breast cancer showed significant rising trend with 2.61% and 1.16% increase per year, respectively. Similar to urban areas, the ASMR of cervical cancer showed significant increase with 7.79% per year from 2006 to 2017.

**Figure 2:**
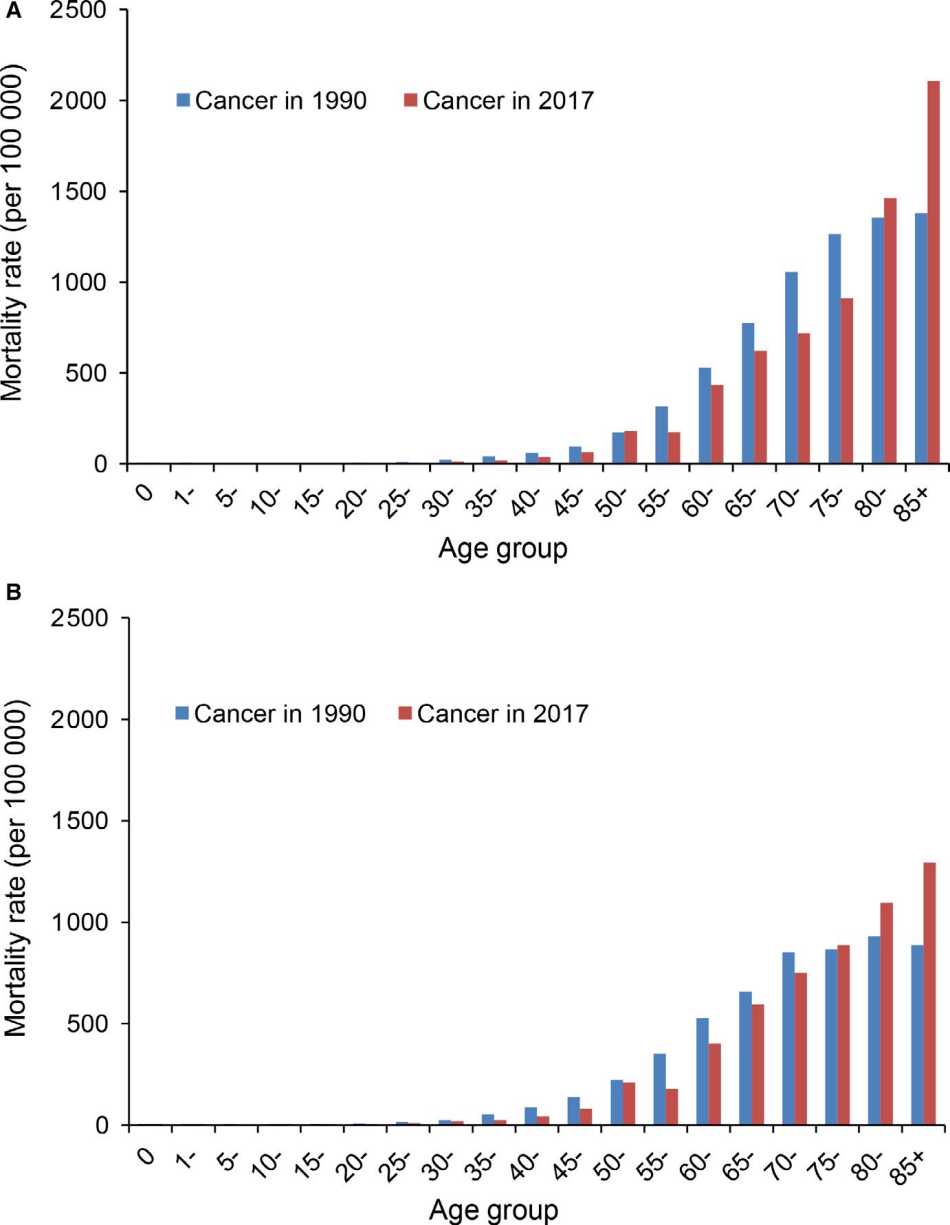
The age‐specific mortality rates of cancer in 1990 and 2017: (A) urban areas, (B) rural areas

Cancer mortality varied greatly among different age groups in the study period (Figure 2). The age‐specific mortality rates of cancers in both urban and rural areas were less than 100 per 100 000 in the group under 45 years old and they gradually enhanced with age. The mortality rates exceeded 800 per 100 000 in the group over 75 years old. In addition, mortality rates in 2017 were higher than those in 1990 for people over 80 years old in urban areas (Figure 2A) and over 75 years old in rural areas (Figure 2B).

As shown in Table 2, the age‐specific mortality rates of all cancers declined among those under 75 years old for both urban and rural residents from 1990 to 2017. However, age‐specific mortality rates significantly increased among people over 80 years old in urban areas and over 75 years old in rural areas. In addition, cancer mortality rates have decreased in urban areas in the 65‐79 age group, whereas cancer mortality rates did not decrease in rural areas but rather increased in the age group of 75‐79 years (Table 2). The trend of age‐specific mortality rate was particularly pronounced for lung cancer (Table S1). The mortality rate of lung cancer significantly increased among those over 50 years old in rural areas and over 80 years old in urban areas during the study period.

**Table 2:** Joinpoint analysis of the age‐specific mortality rates from cancer in urban and rural areas from 1990 to 2017

Age group	Cancer in urban areas		Cancer in rural areas	
	Average APC (%)	95% CI	Average APC (%)	95% CI
0	−0.50	(−1.72, 0.73)	−0.32	(−1.70, 1.09)
1‐4	−0.66	(−1.51, 0.20)	−0.13	(−1.16, 1.43)
5‐9	−1.81*	(−2.49, −1.12)	−1.18*	(−1.81, −0.55)
10‐14	−2.42*	(−3.18, −1.65)	−1.06*	(−1.99, −0.13)
15‐19	−1.19*	(−1.77, −0.60)	−0.15	(−0.94, 0.65)
20‐24	−2.10*	(−3.05, −1.13)	−2.26*	(−3.25, −1.25)
25‐29	−1.42*	(−1.82, −1.03)	−2.41*	(−3.34, −1.47)
30‐34	−1.78*	(−2.27, −1.29)	−2.37*	(−3.30, −1.43)
35‐39	−2.50*	(−3.07, −1.93)	−2.36*	(−3.14, −1.57)
40‐44	−1.84*	(−2.38, −1.29)	−2.14*	(−2.85, −1.43)
45‐49	−1.54*	(−2.37, −0.71)	−2.42*	(−3.23, −1.60)
50‐54	0.29	(−0.16, 0.74)	−0.75*	(−1.33, −0.16)
55‐59	−0.95*	(−1.14, −0.49)	−1.17*	(−1.90, −0.43)
60‐64	−0.96*	(−1.43, −0.49)	−0.65*	(−1.03, −0.26)
65‐69	−1.25*	(−1.64, −0.87)	−0.01	(−0.29, 0.31)
70‐74	−1.53*	(−1.75, −1.30)	−0.04	(−0.38, 0.31)
75‐79	−0.75*	(−1.12, −0.39)	1.21*	(0.72, 1.72)
80‐84	0.62*	(0.28, 0.97)	2.35*	(1.66, 3.05)
85+	2.12*	(1.48, 2.78)	3.65*	(2.45, 4.86)

Abbreviations: APC, annual percent change; CI, confidence interval.

* Significantly difference from zero (*P *< .05).

### 3.2 Trends on YLLs and age‐standardized YLL rates

**Figure 3:**
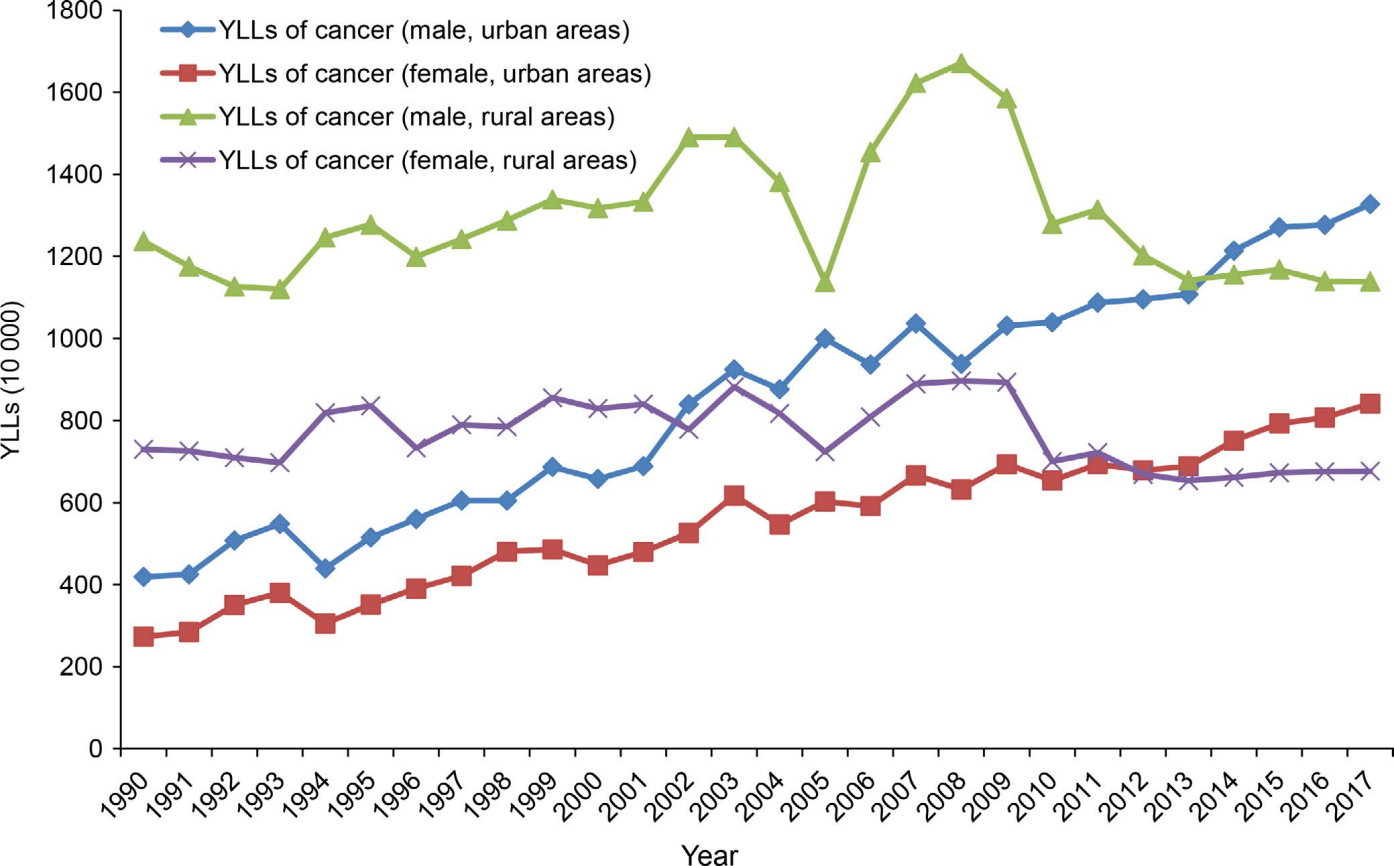
The trends on years of life lost (YLLs) of cancer for male and female in urban and rural areas from 1990 to 2017

Regardless of urban or rural areas, YLLs caused by all cancers were higher in men than those in women (Figure 3). Figure 3 showed a rising trend of YLLs among all urban residents from 1990 to 2017. YLLs in urban areas were 4.19 million for men and 2.73 million for women in 1990, and they rose to 13.27 and 8.41 million in 2017 with 216.71% and 207.87% increase rate, respectively. YLLs in rural areas were 12.37 million for men and 7.30 for women in 1990, they were 11.39 and 6.77 million in 2017 with 7.92% and 7.26% decrease, respectively. YLLs in rural areas were higher than those in urban areas (for both male and female) before 2011, whereas YLLs of urban outstripped those of rural finally with the increase of YLLs in urban areas.

Among urban residents, lung, liver, gastric, and female breast cancer showed an upward trend in YLLs (Figure S1). Among rural residents, YLLs of lung cancer and female breast cancer increased gradually (Figure S2), whereas YLLs of liver and gastric cancer showed a downward trend from 2008 to 2017. In addition, YLLs caused by lung, liver, and gastric cancer for men were higher than those for women in both urban and rural areas.

Table 3 shows age‐standardized YLL rates of cancers among urban and rural residents, as well as APC and 95% CI from 1990 to 2017. The age‐standardized YLL rates of cancer decreased significantly with an average annual decline of 1.02% and 0.85% for urban and rural residents, respectively. The age‐standardized YLL rates of lung, liver, stomach, and breast cancer in urban areas decreased by 0.79%, 1.33%, 2.52%, and 0.46% per year from 1990 to 2017, respectively. Especially, it is worth noting that the age‐standardized YLL rates of lung cancer and breast cancer in rural areas increased with 1.64% and 1.27% per year, respectively.

**Table 3:** Joinpoint analysis of the age‐standardized YLL rates from four main cancers in urban and rural areas from 1990 to 2017

	YLL rate^a^ (1/100 000)		1990‐2017		Period 1		Period 2		Period 3	
	1990	2017	APC (%)	95% CI	Years	APC (%)	Years	APC (%)	Years	APC (%)
Cancer in urban areas	3265.43	2366.41	−1.02*	(−1.20, −0.85)	—					
Lung cancer	772.03	629.42	−0.79*	(−1.03, −0.55)	1990‐2000	0.06	2000‐2004	−4.25	2004‐2017	0.21
Liver cancer	572.03	389.00	−1.33*	(−1.87, −0.79)	—					
Gastric cancer	504.43	229.44	−2.52*	(−2.39, −2.11)	—					
Breast cancer^b^	214.36	206.37	−0.46*	(−0.77, −0.15)	1990‐2000	1.53*	2000‐2003	−5.17	2003‐2017	−0.19
Cancer in rural areas	3442.24	2487.45	−0.85*	(−1.30, −0.39)	—					
Lung cancer	423.38	597.01	1.64*	(1.20, 2.07)	—					
Liver cancer	866.66	489.51	−1.94*	(−2.46, −1.41)	1990‐2003	0.10	2003‐2017	−3.73*		
Gastric cancer	717.98	285.39	−2.93*	(−3.38, −2.48)	1990‐2007	−1.91*	2007‐2017	−5.10*		
Breast cancer^c^	106.84	170.95	1.27*	(0.52, 2.04)	—					

Note

—, No joinpoints.

Abbreviations: YLL, year of life lost; APC, annual percent change; CI, confidence interval.

a Age‐standardized to the Chinese sixth population census in 2010.

b Female breast cancer in urban areas.

c Female breast cancer in rural areas.

* Significantly difference from zero (*P* < .05)

## 4 DISCUSSION

In this study, we assessed the levels and trends of ASMRs and YLLs caused by cancer in urban and rural areas of China from 1990 to 2017. The ASMRs of all cancers among urban residents (both sexes) decreased significantly, such downward trend without significance was also observed among rural residents from 1990 to 2017. Lung, liver, and gastric cancer had the great ASMRs in both urban and rural areas. The age‐specific mortality rates rose with the increase of age in 1990 and 2017. YLLs due to cancer death remained higher level among rural residents and YLLs in urban areas showed a rapidly ascending trend for male and female from 1990 to 2017. The age‐standardized YLL rates decreased significantly in both urban and rural areas during the study period. It is worth noting that the ASMRs and YLL rates of lung and breast cancer in rural areas increased significantly from 1990 to 2017.

In China, all age deaths caused by neoplasm increased by 44.8% between 1990 and 2013, and the ASMR decreased by 17.7%.^8^ We observed significantly decreased trend on ASMR of cancer in urban areas rather than in rural areas. The difference of this decreased trend between urban and rural may be attributed to high education level, better medical service and health‐care affordability, and relatively perfect insurance status in urban areas.^19^, ^20^ However, the survival gap of cancer between urban and rural areas decreased over time with the allocation of more health‐care resources to rural areas by the government.^21^ The ASMRs for men were higher than those for women in both urban and rural areas, which was consistent with previous studies.^22^ This situation might be owing to the proportion of unhealthy lifestyles among men is higher than that of women, such as smoking, drinking, and unhealthy diet structure.^13^, ^23^ Another possible reason may be that men have more opportunities to be exposed to occupational carcinogens.^24^

In terms of age‐specific mortality, the death ages of cancer were mainly concentrated in middle‐aged and elderly population in both areas in 1990 and 2017. The mortality rates of cancer in most age groups showed a downward trend during the study period, except for those over 80 years old in urban areas and those over 75 years old in rural areas, indicating that the age of cancer death is aging. At the same time, it also suggested that the medical conditions in China have improved. However, the number of cancer deaths has increased (1.42 million in 1990 and 2.61 million in 2017) due to the population aging and population growth.^4^

The ASMRs of most cancers in both areas decreased during the study period. We found that the changes in mortality rates varied among different types of cancers. The ASMRs of cancer related to infection factors (liver, gastric, and cervical cancer) declined. However, some cancers related to unhealthy life styles and environmental pollution, such as lung, breast, and colorectal cancer, displayed an upward trend or remained stable. Similar results have been observed in previous study.^25^ The variance among different types of cancer may be related with the change of cancer risk factors such as chronic infection, smoking, low fruit/vegetable intake, and alcohol drinking.^7^, ^13^

We selected main diseases of cancer for further study. Lung, liver, and gastric cancer were the top three leading causes of cancer death, and for women, breast cancer was the most common cancer. Our study indicated that YLLs of lung cancer showed ascending trends in both urban and rural areas from 1990 to 2017, and the ASMR also increased in rural from 1990 to 2017 and in urban during 2004 to 2007. Tobacco smoking and environmental pollutions are considered as major risk factors of lung cancer in China.^10^ Age‐standardized prevalence of daily smoking was 24.2% in China (45.1% for men and 2.1% for women) in 2012, suggesting that cancer levels would be greatly reduced through reducing tobacco consumption among men.^26^ Previous studies suggested that the ascending trends of lung cancer mortality may attributable to the worsening of ambient air pollution.^10^, ^27^ The reason for the difference in lung cancer mortality trends between urban and rural areas might be partly explained by higher smoking rate in rural residents.^28^, ^29^ The ASMRs of liver and gastric cancer have declined significantly in both urban and rural areas from 1990 to 2017. Chronic hepatitis B and C are the main causes of liver cancer deaths, prevention and treatment of which would effectively reduce the incidence and mortality of liver cancer.^30^, ^31^ The prevalence of HBV carriers in China has been greatly reduced after the implementation of the universal HBV vaccination program since 1992.^32^ Treatment for Helicobacter pylori (*H pylori*) infection, which is estimated to cause 65% to 80% of all gastric cancer cases, reduces gastric cancer incidence.^33^, ^34^ Though *H pylori* screening and eradication is likely to be one of the most cost‐effective approaches in gastric cancer prevention, the value of screening mass populations remains controversial.^35^ The ASMR and YLL rate of breast cancer increased significantly in rural areas. The poorer survival rate of breast cancer in rural areas is mainly related to the less access to diagnosis and treatment.^36^

The ASMRs and YLL rates of cancers have decreased in the study period, whereas YLLs were increasing in urban areas or staying higher level in rural areas. The increased burden of cancer might be partly explained by population aging, population growth, and changes in age‐specific rates.^2^, ^37^ The proportion of elderly people (65 years old or older) increased from 5.6% in 1990 to 11.4% in 2017.^38^ Furthermore, population aging is expected to continue in China due to the significant alteration in the age structure caused by past fertility and mortality declines.^39^, ^40^ Other factors related to the burden of cancer include chronic infection, unhealthy lifestyles, environmental, and occupational carcinogens.^24^, ^41^ Some studies showed that it is about between a third and a half of cancers could be preventable, and primary prevention methods are effective ways to fight against cancer, including smoking control, vaccination, and the promotion in physical activity and healthy diet.^2^, ^41^

The strength of this study is that we provide a comprehensive analysis of mortality and YLL rates trends of cancer by joinpoint regression in the past 28 years, and compare the differences between gender, age group, and region. Analyzing the change in mortality trend could assess the efforts of prevention and control measures in cancer. One limitation is that the category of cancer contains ICD‐9 and ICD‐10 in the study period, which may cause incomplete correspondence in results. But a previous study on the comparability of cause of death between ICD‐9 and ICD‐10 indicated that the number of deaths due to malignant neoplasms remained stable across revisions.^42^

## 5 CONCLUSION

In summary, the ASMRs of cancer declined gradually in urban areas, such downward trend without significance was also observed among rural residents in China from 1990 to 2017. It is worth noting that ASMR of lung cancer showed upward trend in rural areas during the study period, this result highlights the importance of tobacco and air pollution control. Though the age‐standardized YLL rates have decreased in both urban and rural areas, YLLs due to cancer death increased continuously in urban areas and remained higher level in rural areas. More works should be done for cancer prevention and control.

## CONFLICTS OF INTEREST

The authors declare no conflict of interest.

## AUTHOR CONTRIBUTIONS

Xingzu Cen, Weiwei Sun, and Weihong Chen designed the research; Xingzu Cen and Dongming Wang performed statistical analysis; Xingzu Cen wrote the paper; Dongming Wang, Weiwei Sun, Limin Cao, Zhuang Zhang, and Bin Wang revised the paper; Weihong Chen had primary responsibility for final content. All authors have read and approved the final manuscript.

## Data Availability

Data and materials of this study are available from the corresponding author upon reasonable request.
