# Supplementary material for: The trends of mortality and years of life lost of cancers in urban and rural areas in China, 1990‐2017
Source: Cancer Med. 2019 Dec 24;9(4):1562–71. doi: 10.1002/cam4.2765 (PMC7013076; doi:10.1002/cam4.2765)
Supplement: Supplementary file 1 [file CAM4-9-1562-s001.doc]

**Table S1.** Joinpoint analysis of the age-specific mortality rates from lung cancer in urban and rural areas from 1990 to 2017.

| Age group | Lung cancer in urban areas | | Lung cancer in rural areas | |
| --- | --- | --- | --- | --- |
|  | Average APC (%) | 95% CI | Average APC (%) | 95% CI |
| 0 | -10.50 | (-21.38, 1.88) | -7.28 | (-19.54, 6.85) |
| 1-4 | -7.64* | (-14.03, -0.79) | -5.91 | (-14.56, 3.60) |
| 5-9 | -2.14 | (-9.08, 5.34) | -4.33 | (-13.63, 5.97) |
| 10-14 | -1.81 | (-10.89, 8.20) | -3.74 | (-12.49, 5.88) |
| 15-19 | -1.27 | (-3.47, 0.99) | 2.08 | (-4.41, 9.02) |
| 20-24 | -2.32* | (-4.29, -0.31) | -1.28 | (-3.59, 1.08) |
| 25-29 | -1.94* | (-2.88, -0.99) | -1.56 | (-3.36, 0.27) |
| 30-34 | -1.75* | (-2.35, -1.14) | -1.04* | (-1.99, -0.08) |
| 35-39 | -2.54* | (-3.06, -2.01) | 0.50 | (-1.55, 0.57) |
| 40-44 | -1.67* | (-2.31, -1.03) | 0.01 | (-1.01, 1.04) |
| 45-49 | -1.42* | (-2.23, -0.60) | -0.06 | (-1.01, 0.90) |
| 50-54 | 0.49* | (0.11, 0.87) | 1.61* | (0.95, 2.26) |
| 55-59 | -0.91* | (-1.39, -0.42) | 1.02* | (0.35, 1.70) |
| 60-64 | -1.08* | (-1.78, -0.36) | 1.52* | (1.03, 2.01) |
| 65-69 | -1.31* | (-1.84, -0.77) | 2.42* | (2.00, 2.83) |
| 70-74 | -1.21* | (-1.58, -0.85) | 2.69* | (2.28, 3.09) |
| 75-79 | 0.06 | (-0.43, 0.55) | 4.37* | (3.75, 5.00) |
| 80-84 | 1.81* | (1.37, 2.26) | 5.83* | (5.05, 6.62) |
| 85+ | 3.74* | (3.06, 4.43) | 7.30* | (6.12, 8.48) |

* Significantly difference from zero (*P*<0.05). Abbreviations: APC, annual percent change; CI, confidence interval.


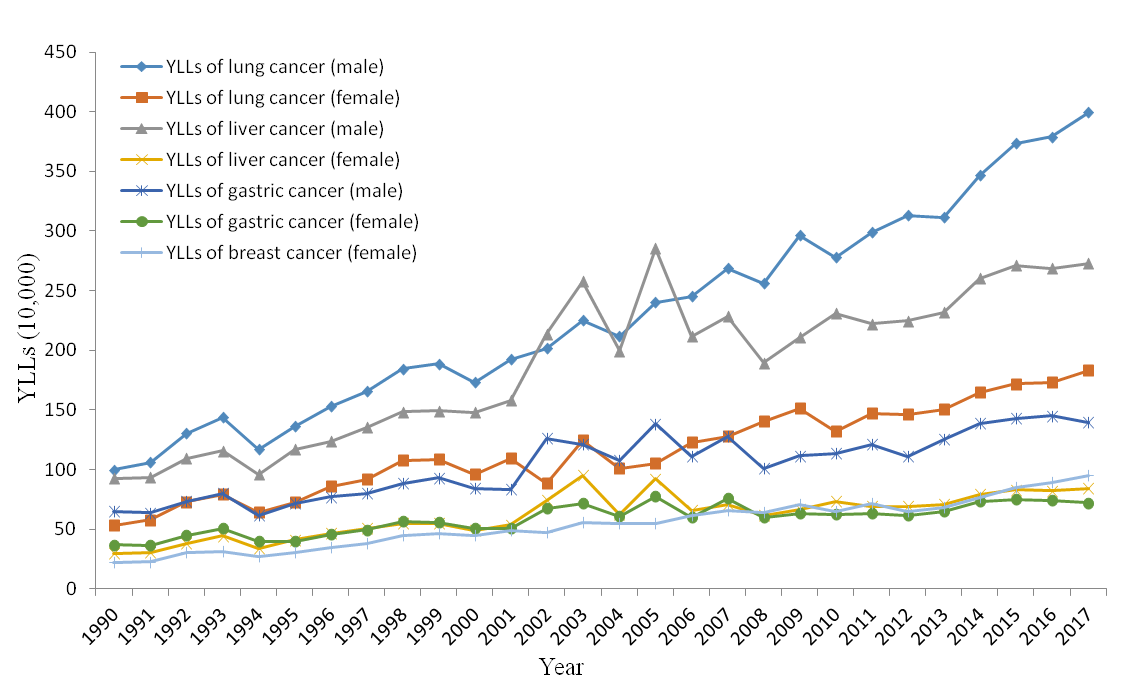


**Figure S1.** The trends on years of life lost (YLLs) of lung cancer, liver cancer, gastric cancer and female breast cancer in urban areas from 1990 to 2017.


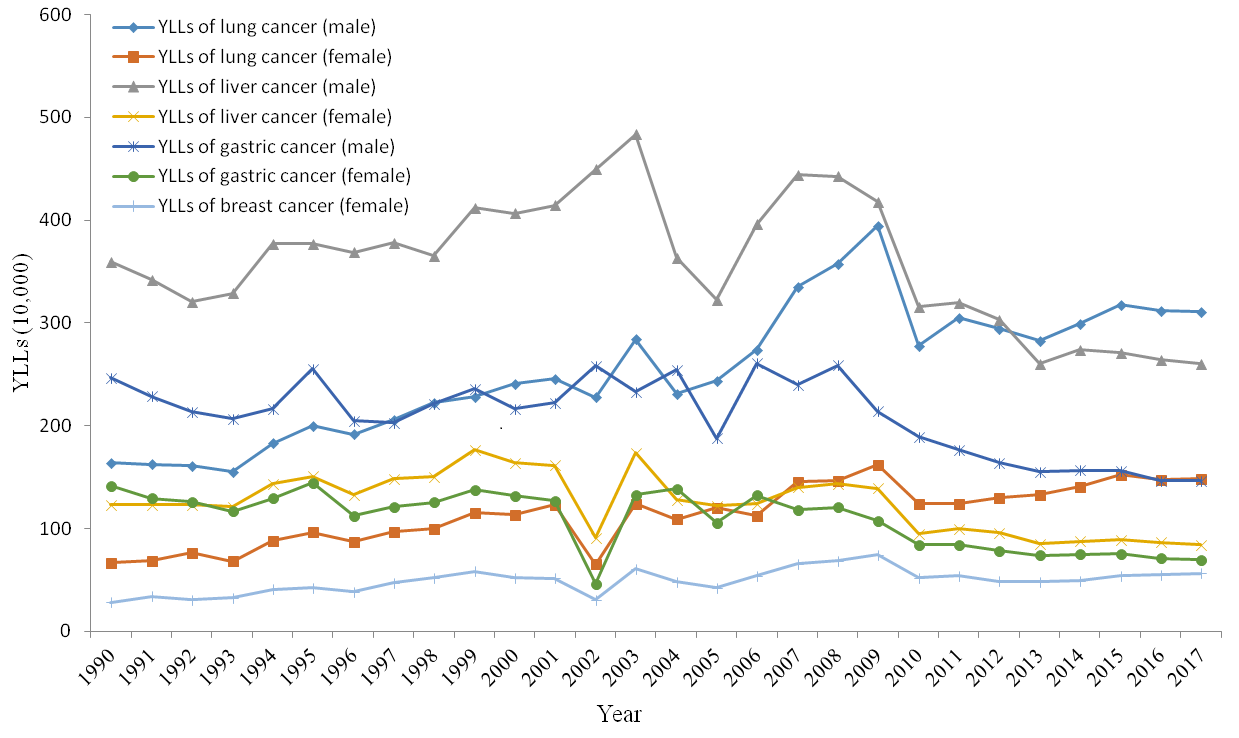


**Figure S2.** The trends on years of life lost (YLLs) of lung cancer, liver cancer, gastric cancer and female breast cancer in rural areas from 1990 to 2017.
